# Primary angiitis of the central nervous system in children - case series

**DOI:** 10.1186/1546-0096-9-S1-P94

**Published:** 2011-09-14

**Authors:** V Krakovská, R Stibitzová, T Pískovský, Z Vrábelová, M Macků, D Němcová, P Doležalová

**Affiliations:** 1 Paediatric Rheumatology Unit, General University Hospital in Prague, Czech Republic; 2 Department of Paediatric Neurology, Thomayer University Hospital in Prague, Czech Republic; 3 Department of Paediatrics, Teaching Hospital in Ostrava, Czech Republic; 4 Department of Paediatric Neurology, University Hospital Motol, Prague, Czech Republic; 5 Department of Paediatrics, University Hospital in Brno, Czech Republic

## Background

Primary angiitis of the central nervous system (PACNS) is a rare inflammatory brain disease with variable clinical manifestations. Only limited data exist with regard to efficient diagnostic and therapeutic algorithms and disease outcomes in children.

## Aim

To characterize clinical features, neuroimaging findings, treatment and disease outcome in a small cohort of Czech children with PACNS.

## Methods

A retrospective chart review of patients referred for suspected PACNS was performed. MRI lesions detected at disease onset were classified as unilateral (UL), bilateral (BL), unifocal (F), multifocal (MF), angiography (MRA and/or conventional) positive (medium/large vessel disease) or negative (small vessel disease). According to the evolution of clinical manifestations and neuroimaging PACNS was classified as progressive or non-progressive. Data on treatment and follow-up were collected.

## Results

Seven female patients (all Caucasian, mean age 14,8 years, median F/U 2 years) were identified (Table). Three had favourable outcome. Four (1,2,5,7) had progressive disease resulting in significant impairment. Their mean interval from onset to diagnosis was 7 months. All 4 had multifocal lesions, 2 had negative angiography, 3 did not receive any anti-inflammatory therapy early in the disease course.

**Table:** 

Patient	1	2	3	4	5	6	7
**Age at onset (years)**	13	1,5	12	16	5,5	6	15
**Time to diagnosis (months)**	30	5	1	1,5	12	1	1,5
**F/U (years)**	4	13	5	0,5	2	1,5	1
**Manifestation at onset**	generalised seizures	focal seizure, stroke	seizure sensory deficit	sensory deficit, stroke	stroke	stroke with ataxia	seizures stroke
**MRI at onset**	UL, MF	UL, MF	BL, MF	BL,MF	UL,MF	UL, F	UL,MF
**Angiography at onset**	N/D	posit.	posit.	posit.	posit.	posit.	neg.
**Initial therapy**	AED	AED	ACA	ACA, CS	0	ACA	IVMP, AED
**Manifestation at F/U**	seizures cognitive dysfunction	hemiparesis developmental delay	normal	normal	cognitive dysfunction, hemiparesis	normal	organic psychosis
**MRI at F/U**	BL, MF	N/D	N/D	N/D	BL, MF	regression	BL, MF
**Angiography F/U**	neg.	N/D	N/D	N/D	regression	regression	neg.
**Therapy at F/U**	AED, CS	AED, ACA	ACA	CS	ACA	ACA	CYC, CS

ACA - acetylsalicylic acid, AED - anti-epileptic drugs, CS - oral corticosteroid (prednisone or methylprednisolone), CYC - cyclophosphamide, IVMP- intravenous methylprednisolone, N/D- not done

## Conclusion

Unfavourable outcome of this patient cohort reflects potential severity as well as low physicians awareness of PACNS resulting in diagnostic delay and insufficient therapy. A working group of paediatric specialists is being formed in the Czech Republic in order to establish diagnostic and therapeutic algorithms in close interdisciplinary and multicenter collaboration.

